# Supplementary material for: Algorithmic encoding of protected characteristics in chest X-ray disease detection models
Source: eBioMedicine. 2023 Feb 13;89:104467. doi: 10.1016/j.ebiom.2023.104467 (PMC10025760; doi:10.1016/j.ebiom.2023.104467)
Supplement: Supplementary material A [file mmc1.docx]

# Supplementary material A: Implementation details

For the deep neural network models, we use a PyTorch implementation of a DenseNet-121.^1^ All chest X-ray images are resized using bi-linear interpolation to fit the required 224 x 224 pixel resolution. For the models trained for disease detection, race and sex classification, we initialize the network backbone with pre-trained ImageNet weights as provided in the torchvision module, except for the SPLIT models using backbones with random weights. We employ the Adam optimizer with default parameters and a learning rate of 0·001 for all our experiments.^2^ We use the same configuration when training the prediction layers in the SPLIT experiments. We select the model checkpoint with the lowest cross-entropy loss on the validation set. To assess the variation in model performance due to randomness, we have trained the main disease detection models three times with different random seeds. The standard deviation across the reported performance metrics was 0·006 ± 0·005 on CheXpert and 0·004 ± 0·003 on MIMIC-CXR. We also explored another network architecture, ResNet-34^3^, with the corresponding results reported in Tables S1, S6, S7, and Figure S3. Additional implementation details can be found in our code repository, including all trained models and scripts for test-set resampling and performance evaluation.

The input to PCA is a n-by-m feature matrix where n is the number of scans in the test set (38,240 for CheXpert and 55,262 for MIMIC-CXR), and m is the size of the output of the penultimate layer in the neural network (which is 1,024 for DenseNet-121 and 512 for ResNet-34). We use the PCA and t-SNE implementations provided in the Python-based machine learning library scikit-learn.^4^ We initialize t-SNE with PCA embeddings as this has been shown to yield more consistent results.^5^ We use PCA embeddings that preserve 99% of the variance of the feature representations. Otherwise, we use the default parameters for t-SNE. To improve the visibility in the scatter plots, we randomly sample 1,000 scans from each racial group, so in total 3,000 samples are shown in each scatter plot. PCA and t-SNE were computed on the entire test sets to capture the full variation in the subgroups. The random samples are used for visualization purposes only. For statistical testing, we use the two-sample Kolmogorov-Smirnov test from SciPy 1.8.0. The p-values are adjusted for multiple testing using the Benjamini-Yekutieli procedure and significance is determined at a 95% confidence level.^6^

**References**

1 Huang G, Liu Z, Van Der Maaten L, Weinberger KQ. Densely connected convolutional networks. In: Proceedings of the IEEE Conference on Computer Vision and Pattern Recognition. 2017: 4700–8.

2 Kingma DP, Ba J. Adam: A Method for Stochastic Optimization. In: Proceedings of the 3rd International Conference on Learning Representations. 2015.

3 He K, Zhang X, Ren S, Sun J. Deep residual learning for image recognition. In: Proceedings of the IEEE Conference on Computer Vision and Pattern Recognition. 2016: 770–8.

4 Pedregosa F, Varoquaux G, Gramfort A, et al. Scikit-learn: Machine learning in Python. Journal of Machine Learning Research 2011; 12: 2825–30.

5 Kobak D, Berens P. The art of using t-SNE for single-cell transcriptomics. Nature Communications 2019; 10: 5416.

6 Benjamini Y, Yekutieli D. The Control of the False Discovery Rate in Multiple Testing under Dependency. Ann Stat 2001; 29: 1165–88.
