# Supplementary material for: Algorithmic encoding of protected characteristics in chest X-ray disease detection models
Source: eBioMedicine. 2023 Feb 13;89:104467. doi: 10.1016/j.ebiom.2023.104467 (PMC10025760; doi:10.1016/j.ebiom.2023.104467)
Supplement: Supplementary Tables S1–S7 [file mmc2.docx]

# Supplementary material B: Additional tables

Table S1. Disease detection with ResNet-34 trained and tested on CheXpert

|  | **No finding** | | | | |
| --- | --- | --- | --- | --- | --- |
|  | White | Asian | Black | Female | Male |
| Test-set | AUC (95% CI) | | | | |
| Original | 0·87 (0·86-0·88) | 0·87 (0·85-0·88) | 0·87 (0·85-0·89) | 0·86 (0·85-0·87) | 0·87 (0·86-0·88) |
| Resampled | 0·87 (0·86-0·87) | 0·86 (0·86-0·87) | 0·87 (0·87-0·88) | 0·85 (0·85-0·86) | 0·88 (0·88-0·89) |
| Multitask | 0·86 (0·85-0·87) | 0·86 (0·85-0·88) | 0·87 (0·85-0·89) | 0·86 (0·85-0·87) | 0·86 (0·86-0·87) |
|  | TPR (95% CI) | | | | |
| Original | 0·79 (0·77-0·81) | 0·80 (0·77-0·84) | 0·79 (0·75-0·84) | 0·78 (0·75-0·80) | 0·81 (0·79-0·83) |
| Resampled | 0·79 (0·78-0·81) | 0·81 (0·79-0·82) | 0·77 (0·75-0·78) | 0·75 (0·74-0·77) | 0·82 (0·81-0·83) |
| Multitask | 0·76 (0·75-0·78) | 0·82 (0·79-0·86) | 0·84 (0·81-0·89) | 0·74 (0·72-0·77) | 0·81 (0·79-0·83) |
|  | FPR (95% CI) | | | | |
| Original | 0·20 (0·20-0·20) | 0·19 (0·18-0·20) | 0·21 (0·20-0·23) | 0·20 (0·20-0·21) | 0·20 (0·19-0·20) |
| Resampled | 0·21 (0·20-0·21) | 0·20 (0·20-0·20) | 0·19 (0·19-0·20) | 0·20 (0·20-0·20) | 0·20 (0·20-0·20) |
| Multitask | 0·19 (0·19-0·19) | 0·24 (0·23-0·25) | 0·25 (0·23-0·26) | 0·17 (0·17-0·18) | 0·22 (0·21-0·22) |
|  | Youden’s J statistic (95% CI) | | | | |
| Original | 0·59 (0·57-0·61) | 0·61 (0·57-0·65) | 0·58 (0·53-0·63) | 0·57 (0·55-0·60) | 0·61 (0·59-0·63) |
| Resampled | 0·59 (0·57-0·60) | 0·61 (0·59-0·62) | 0·58 (0·56-0·59) | 0·56 (0·54-0·57) | 0·62 (0·61-0·63) |
| Multitask | 0·57 (0·56-0·59) | 0·59 (0·55-0·62) | 0·59 (0·56-0·64) | 0·57 (0·55-0·59) | 0·59 (0·57-0·61) |
|  | **Pleural effusion** | | | | |
|  | White | Asian | Black | Female | Male |
| Test-set | AUC (95% CI) | | | | |
| Original | 0·86 (0·86-0·86) | 0·87 (0·87-0·88) | 0·85 (0·84-0·87) | 0·86 (0·86-0·87) | 0·86 (0·86-0·86) |
| Resampled | 0·86 (0·86-0·86) | 0·88 (0·87-0·88) | 0·84 (0·83-0·84) | 0·86 (0·86-0·86) | 0·85 (0·85-0·86) |
| Multitask | 0·86 (0·85-0·86) | 0·88 (0·87-0·88) | 0·86 (0·84-0·87) | 0·86 (0·86-0·87) | 0·86 (0·86-0·86) |
|  | TPR (95% CI) | | | | |
| Original | 0·76 (0·75-0·77) | 0·76 (0·74-0·78) | 0·68 (0·64-0·71) | 0·76 (0·75-0·77) | 0·76 (0·75-0·77) |
| Resampled | 0·77 (0·76-0·78) | 0·79 (0·78-0·79) | 0·68 (0·67-0·69) | 0·75 (0·74-0·76) | 0·74 (0·73-0·75) |
| Multitask | 0·76 (0·75-0·77) | 0·79 (0·77-0·81) | 0·68 (0·65-0·71) | 0·77 (0·76-0·78) | 0·75 (0·74-0·76) |
|  | FPR (95% CI) | | | | |
| Original | 0·21 (0·20-0·21) | 0·19 (0·17-0·20) | 0·17 (0·15-0·19) | 0·20 (0·20-0·21) | 0·20 (0·19-0·20) |
| Resampled | 0·21 (0·20-0·21) | 0·20 (0·19-0·20) | 0·20 (0·19-0·20) | 0·21 (0·20-0·21) | 0·19 (0·19-0·20) |
| Multitask | 0·21 (0·20-0·21) | 0·20 (0·19-0·21) | 0·15 (0·13-0·16) | 0·21 (0·20-0·21) | 0·19 (0·19-0·20) |
|  | Youden’s J statistic (95% CI) | | | | |
| Original | 0·56 (0·55-0·57) | 0·58 (0·55-0·60) | 0·51 (0·47-0·54) | 0·56 (0·54-0·57) | 0·56 (0·55-0·57) |
| Resampled | 0·56 (0·55-0·57) | 0·59 (0·58-0·60) | 0·49 (0·47-0·49) | 0·54 (0·53-0·55) | 0·55 (0·54-0·55) |
| Multitask | 0·56 (0·55-0·56) | 0·59 (0·57-0·61) | 0·53 (0·49-0·56) | 0·56 (0·55-0·57) | 0·56 (0·55-0·57) |

Disease detection results reported separately for each race group and biological sex for ‘no finding’ (top) and ‘pleural effusion’ (bottom). TPR and FPR in subgroups are determined using a fixed decision threshold optimized over the whole patient population for a target FPR of 0·20.

Table S2. SPLIT for race classification with DenseNet-121 on CheXpert

|  | White | Asian | Black |
| --- | --- | --- | --- |
| Neural network backbone | AUC (95% CI) | | |
| Random weights | 0·70 (0·70-0·71) | 0·75 (0·74-0·76) | 0·67 (0·66-0·68) |
| ImageNet | 0·77 (0·76-0·77) | 0·79 (0·79-0·80) | 0·78 (0·77-0·79) |
| Disease detection (all patients) | 0·78 (0·77-0·79) | 0·80 (0·80-0·81) | 0·78 (0·77-0·79) |
| Disease detection (White patients) | 0·77 (0·76-0·77) | 0·79 (0·79-0·80) | 0·79 (0·78-0·80) |
| Race classification (single task) | 0·96 (0·96-0·96) | 0·96 (0·96-0·97) | 0·97 (0·96-0·97) |
| Race classification (multitask) | 0·95 (0·95-0·96) | 0·96 (0·95-0·96) | 0·96 (0·96-0·97) |
|  | TPR (95% CI) | | |
| Random weights | 0·64 (0·58-0·67) | 0·73 (0·66-0·78) | 0·64 (0·56-0·69) |
| ImageNet | 0·72 (0·65-0·73) | 0·76 (0·71-0·79) | 0·69 (0·66-0·73) |
| Disease detection (all patients) | 0·69 (0·67-0·72) | 0·73 (0·72-0·76) | 0·70 (0·62-0·73) |
| Disease detection (White patients) | 0·70 (0·65-0·75) | 0·71 (0·67-0·76) | 0·69 (0·67-0·77) |
| Race classification (single task) | 0·89 (0·89-0·91) | 0·90 (0·88-0·91) | 0·90 (0·89-0·91) |
| Race classification (multitask) | 0·89 (0·89-0·90) | 0·88 (0·87-0·90) | 0·90 (0·88-0·92) |
|  | FPR (95% CI) | | |
| Random weights | 0·34 (0·28-0·37) | 0·35 (0·28-0·40) | 0·39 (0·31-0·43) |
| ImageNet | 0·32 (0·25-0·34) | 0·31 (0·26-0·34) | 0·28 (0·25-0·30) |
| Disease detection (all patients) | 0·27 (0·25-0·30) | 0·27 (0·26-0·30) | 0·28 (0·21-0·31) |
| Disease detection (White patients) | 0·30 (0·25-0·36) | 0·27 (0·23-0·32) | 0·26 (0·25-0·33) |
| Race classification (single task) | 0·10 (0·09-0·12) | 0·08 (0·08-0·10) | 0·08 (0·08-0·09) |
| Race classification (multitask) | 0·11 (0·11-0·13) | 0·09 (0·08-0·11) | 0·09 (0·08-0·11) |
|  | Youden’s J statistic (95% CI) | | |
| Random weights | 0·30 (0·29-0·31) | 0·38 (0·37-0·39) | 0·25 (0·24-0·27) |
| ImageNet | 0·40 (0·39-0·41) | 0·45 (0·44-0·46) | 0·41 (0·40-0·43) |
| Disease detection (all patients) | 0·42 (0·41-0·43) | 0·46 (0·45-0·47) | 0·42 (0·40-0·44) |
| Disease detection (White patients) | 0·40 (0·39-0·41) | 0·44 (0·43-0·46) | 0·43 (0·42-0·45) |
| Race classification (single task) | 0·79 (0·79-0·80) | 0·81 (0·80-0·82) | 0·82 (0·81-0·83) |
| Race classification (multitask) | 0·78 (0·77-0·78) | 0·79 (0·78-0·80) | 0·81 (0·80-0·82) |

Performance is determined in a one-vs-rest approach for each racial group. TPR and FPR in subgroups are determined using a fixed decision threshold optimized over the whole patient population for highest Youden’s J statistic. For comparison, we also report the performance for single task and multitask neural networks trained specifically for classifying race, confirming the high accuracy reported in the literature.[^8^](https://paperpile.com/c/d9G4RW/ypQ7s)

Table S3. SPLIT for sex classification with DenseNet-121 on CheXpert

|  | White | Asian | Black |
| --- | --- | --- | --- |
| Neural network backbone | AUC (95% CI) | | |
| Random weights | 0·86 (0·86-0·86) | 0·82 (0·80-0·83) | 0·87 (0·86-0·88) |
| ImageNet | 0·92 (0·92-0·93) | 0·91 (0·90-0·91) | 0·93 (0·92-0·94) |
| Disease detection (all patients) | 0·95 (0·94-0·95) | 0·91 (0·90-0·92) | 0·94 (0·93-0·95) |
| Disease detection (male patients) | 0·94 (0·94-0·95) | 0·91 (0·90-0·91) | 0·93 (0·92-0·94) |
| Sex classification (single task) | 1·00 (1·00-1·00) | 1·00 (1·00-1·00) | 1·00 (1·00-1·00) |
| Sex classification (multitask) | 1·00 (1·00-1·00) | 1·00 (1·00-1·00) | 1·00 (1·00-1·00) |
|  | TPR (95% CI) | | |
| Random weights | 0·79 (0·76-0·82) | 0·74 (0·70-0·83) | 0·85 (0·81-0·88) |
| ImageNet | 0·86 (0·84-0·88) | 0·85 (0·80-0·87) | 0·86 (0·83-0·89) |
| Disease detection (all patients) | 0·90 (0·87-0·90) | 0·80 (0·78-0·85) | 0·86 (0·82-0·90) |
| Disease detection (male patients) | 0·87 (0·86-0·89) | 0·85 (0·79-0·86) | 0·86 (0·82-0·89) |
| Sex classification (single task) | 0·98 (0·98-0·99) | 0·99 (0·98-0·99) | 0·98 (0·97-0·99) |
| Sex classification (multitask) | 0·98 (0·98-0·99) | 0·97 (0·97-0·99) | 0·99 (0·99-1·00) |
|  | FPR (95% CI) | | |
| Random weights | 0·23 (0·20-0·26) | 0·25 (0·21-0·34) | 0·26 (0·21-0·29) |
| ImageNet | 0·17 (0·15-0·19) | 0·20 (0·15-0·22) | 0·16 (0·12-0·19) |
| Disease detection (all patients) | 0·14 (0·11-0·15) | 0·13 (0·12-0·19) | 0·12 (0·08-0·16) |
| Disease detection (male patients) | 0·13 (0·12-0·14) | 0·19 (0·13-0·20) | 0·15 (0·11-0·18) |
| Sex classification (single task) | 0·02 (0·01-0·02) | 0·03 (0·02-0·03) | 0·02 (0·01-0·04) |
| Sex classification (multitask) | 0·01 (0·01-0·02) | 0·02 (0·01-0·03) | 0·01 (0·00-0·02) |
|  | Youden’s J statistic (95% CI) | | |
| Random weights | 0·56 (0·55-0·57) | 0·49 (0·47-0·51) | 0·59 (0·57-0·62) |
| ImageNet | 0·69 (0·68-0·70) | 0·65 (0·64-0·68) | 0·70 (0·68-0·73) |
| Disease detection (all patients) | 0·76 (0·75-0·76) | 0·66 (0·65-0·69) | 0·74 (0·72-0·77) |
| Disease detection (male patients) | 0·75 (0·74-0·75) | 0·66 (0·64-0·68) | 0·71 (0·68-0·74) |
| Sex classification (single task) | 0·97 (0·96-0·97) | 0·96 (0·96-0·97) | 0·96 (0·95-0·97) |
| Sex classification (multitask) | 0·97 (0·97-0·97) | 0·95 (0·95-0·96) | 0·98 (0·98-0·99) |

Performance for classifying sex reported separately for each racial group. TPR and FPR in subgroups are determined using a fixed decision threshold optimized over the whole patient population for highest Youden’s J statistic. For comparison, we also report the performance for single task and multitask neural networks trained specifically for sex classification, confirming high accuracy reported in the literature.[^7^](https://paperpile.com/c/d9G4RW/SIlro)

Table S4. SPLIT for race classification with DenseNet-121 on MIMIC-CXR

|  | White | Asian | Black |
| --- | --- | --- | --- |
| Neural network backbone | AUC (95% CI) | | |
| Random weights | 0·67 (0·67-0·68) | 0·73 (0·72-0·74) | 0·68 (0·67-0·68) |
| ImageNet | 0·77 (0·77-0·78) | 0·79 (0·78-0·80) | 0·78 (0·77-0·78) |
| Disease detection (all patients) | 0·80 (0·79-0·80) | 0·77 (0·76-0·78) | 0·81 (0·81-0·82) |
| Disease detection (White patients) | 0·79 (0·78-0·79) | 0·77 (0·76-0·78) | 0·80 (0·79-0·80) |
| Race classification (single task) | 0·97 (0·97-0·98) | 0·97 (0·97-0·97) | 0·98 (0·98-0·98) |
| Race classification (multitask) | 0·97 (0·96-0·97) | 0·96 (0·96-0·96) | 0·97 (0·97-0·97) |
|  | TPR (95% CI) | | |
| Random weights | 0·66 (0·65-0·68) | 0·74 (0·66-0·78) | 0·59 (0·55-0·62) |
| ImageNet | 0·74 (0·68-0·76) | 0·76 (0·65-0·79) | 0·68 (0·66-0·72) |
| Disease detection (all patients) | 0·75 (0·72-0·79) | 0·71 (0·61-0·75) | 0·71 (0·69-0·73) |
| Disease detection (White patients) | 0·72 (0·69-0·75) | 0·69 (0·67-0·75) | 0·68 (0·67-0·73) |
| Race classification (single task) | 0·93 (0·91-0·94) | 0·89 (0·87-0·91) | 0·92 (0·91-0·93) |
| Race classification (multitask) | 0·92 (0·91-0·93) | 0·89 (0·87-0·91) | 0·90 (0·89-0·91) |
|  | FPR (95% CI) | | |
| Random weights | 0·41 (0·39-0·43) | 0·40 (0·32-0·44) | 0·34 (0·30-0·36) |
| ImageNet | 0·33 (0·27-0·36) | 0·33 (0·22-0·35) | 0·27 (0·24-0·30) |
| Disease detection (all patients) | 0·31 (0·27-0·34) | 0·30 (0·21-0·35) | 0·24 (0·22-0·26) |
| Disease detection (White patients) | 0·29 (0·27-0·33) | 0·28 (0·26-0·34) | 0·24 (0·23-0·29) |
| Race classification (single task) | 0·10 (0·08-0·10) | 0·06 (0·06-0·08) | 0·07 (0·06-0·08) |
| Race classification (multitask) | 0·11 (0·10-0·13) | 0·11 (0·09-0·13) | 0·08 (0·07-0·09) |
|  | Youden’s J statistic (95% CI) | | |
| Random weights | 0·25 (0·25-0·26) | 0·34 (0·32-0·36) | 0·26 (0·25-0·27) |
| ImageNet | 0·41 (0·40-0·42) | 0·43 (0·41-0·45) | 0·42 (0·41-0·43) |
| Disease detection (all patients) | 0·45 (0·44-0·46) | 0·40 (0·39-0·43) | 0·47 (0·46-0·48) |
| Disease detection (White patients) | 0·42 (0·42-0·43) | 0·41 (0·39-0·43) | 0·45 (0·44-0·46) |
| Race classification (single task) | 0·83 (0·83-0·84) | 0·83 (0·81-0·84) | 0·85 (0·84-0·85) |
| Race classification (multitask) | 0·81 (0·80-0·81) | 0·78 (0·77-0·80) | 0·82 (0·82-0·83) |

Performance is determined in a one-vs-rest approach for each racial group. TPR and FPR in subgroups are determined using a fixed decision threshold optimized over the whole patient population for highest Youden’s J statistic. For comparison, we also report the performance for single task and multitask neural networks trained specifically for classifying race, confirming the high accuracy reported in the literature.[^8^](https://paperpile.com/c/d9G4RW/ypQ7s)

Table S5. SPLIT for sex classification with DenseNet-121 on MIMIC-CXR

|  | White | Asian | Black |
| --- | --- | --- | --- |
| Neural network backbone | AUC (95% CI) | | |
| Random weights | 0·85 (0·85-0·86) | 0·81 (0·79-0·83) | 0·87 (0·86-0·88) |
| ImageNet | 0·92 (0·92-0·92) | 0·89 (0·88-0·90) | 0·92 (0·92-0·93) |
| Disease detection (all patients) | 0·94 (0·94-0·94) | 0·92 (0·91-0·93) | 0·94 (0·94-0·95) |
| Disease detection (male patients) | 0·93 (0·93-0·93) | 0·89 (0·88-0·91) | 0·94 (0·93-0·94) |
| Sex classification (single task) | 1·00 (1·00-1·00) | 1·00 (1·00-1·00) | 1·00 (1·00-1·00) |
| Sex classification (multitask) | 1·00 (1·00-1·00) | 1·00 (1·00-1·00) | 1·00 (1·00-1·00) |
|  | TPR (95% CI) | | |
| Random weights | 0·77 (0·75-0·81) | 0·84 (0·78-0·87) | 0·77 (0·75-0·83) |
| ImageNet | 0·84 (0·83-0·86) | 0·79 (0·76-0·86) | 0·84 (0·82-0·87) |
| Disease detection (all patients) | 0·86 (0·85-0·89) | 0·84 (0·78-0·89) | 0·88 (0·85-0·90) |
| Disease detection (male patients) | 0·85 (0·83-0·87) | 0·82 (0·75-0·87) | 0·87 (0·84-0·89) |
| Sex classification (single task) | 0·98 (0·98-0·99) | 0·97 (0·96-0·98) | 0·98 (0·98-0·99) |
| Sex classification (multitask) | 0·98 (0·98-0·98) | 0·98 (0·96-0·99) | 0·98 (0·97-0·99) |
|  | FPR (95% CI) | | |
| Random weights | 0·23 (0·21-0·27) | 0·35 (0·28-0·37) | 0·20 (0·18-0·26) |
| ImageNet | 0·16 (0·15-0·17) | 0·17 (0·14-0·23) | 0·15 (0·12-0·18) |
| Disease detection (all patients) | 0·14 (0·13-0·17) | 0·15 (0·10-0·20) | 0·14 (0·11-0·16) |
| Disease detection (male patients) | 0·15 (0·13-0·17) | 0·19 (0·11-0·23) | 0·15 (0·12-0·17) |
| Sex classification (single task) | 0·01 (0·01-0·02) | 0·01 (0·01-0·03) | 0·01 (0·01-0·02) |
| Sex classification (multitask) | 0·02 (0·01-0·02) | 0·02 (0·01-0·03) | 0·02 (0·01-0·03) |
|  | Youden’s J statistic (95% CI) | | |
| Random weights | 0·54 (0·53-0·55) | 0·49 (0·46-0·53) | 0·57 (0·56-0·59) |
| ImageNet | 0·68 (0·67-0·69) | 0·62 (0·59-0·66) | 0·69 (0·68-0·71) |
| Disease detection (all patients) | 0·72 (0·71-0·73) | 0·69 (0·66-0·72) | 0·74 (0·73-0·76) |
| Disease detection (male patients) | 0·70 (0·69-0·70) | 0·63 (0·61-0·67) | 0·72 (0·71-0·73) |
| Sex classification (single task) | 0·97 (0·97-0·97) | 0·96 (0·94-0·97 | 0·97 (0·96-0·97) |
| Sex classification (multitask) | 0·96 (0·96-0·96) | 0·96 (0·95-0·97) | 0·96 (0·95-0·96) |

Performance for classifying sex reported separately for each racial group. TPR and FPR in subgroups are determined using a fixed decision threshold optimized over the whole patient population for highest Youden’s J statistic. For comparison, we also report the performance for single task and multitask neural networks trained specifically for sex classification, confirming high accuracy reported in the literature.[^7^](https://paperpile.com/c/d9G4RW/SIlro)

Table S6. SPLIT for race classification with ResNet-34 on CheXpert

|  | White | Asian | Black |
| --- | --- | --- | --- |
| Neural network backbone | AUC (95% CI) | | |
| Random weights | 0·60 (0·60-0·61) | 0·65 (0·64-0·65) | 0·58 (0·57-0·59) |
| ImageNet | 0·73 (0·73-0·74) | 0·77 (0·77-0·78) | 0·73 (0·72-0·74) |
| Disease detection (all patients) | 0·74 (0·73-0·74) | 0·76 (0·75-0·76) | 0·76 (0·75-0·77) |
| Disease detection (White patients) | 0·72 (0·72-0·73) | 0·75 (0·75-0·76) | 0·74 (0·72-0·75) |
| Race classification (single task) | 0·95 (0·95-0·96) | 0·96 (0·95-0·96) | 0·96 (0·96-0·97) |
| Race classification (multitask) | 0·95 (0·95-0·95) | 0·95 (0·95-0·96) | 0·96 (0·95-0·96) |
|  | TPR (95% CI) | | |
| Random weights | 0·54 (0·46-0·62) | 0·68 (0·63-0·71) | 0·56 (0·35-0·60) |
| ImageNet | 0·63 (0·62-0·67) | 0·69 (0·68-0·76) | 0·71 (0·59-0·75) |
| Disease detection (all patients) | 0·66 (0·61-0·70) | 0·72 (0·66-0·80) | 0·75 (0·66-0·76) |
| Disease detection (White patients) | 0·65 (0·60-0·69) | 0·70 (0·67-0·78) | 0·63 (0·58-0·68) |
| Race classification (single task) | 0·89 (0·89-0·91) | 0·87 (0·86-0·89) | 0·89 (0·88-0·92) |
| Race classification (multitask) | 0·87 (0·86-0·89) | 0·87 (0·86-0·90) | 0·88 (0·86-0·90) |
|  | FPR (95% CI) | | |
| Random weights | 0·39 (0·30-0·47) | 0·45 (0·41-0·49) | 0·44 (0·23-0·47) |
| ImageNet | 0·29 (0·28-0·33) | 0·28 (0·27-0·34) | 0·37 (0·24-0·40) |
| Disease detection (all patients) | 0·31 (0·25-0·34) | 0·33 (0·27-0·42) | 0·36 (0·28-0·37) |
| Disease detection (White patients) | 0·32 (0·27-0·36) | 0·32 (0·28-0·39) | 0·28 (0·23-0·32) |
| Race classification (single task) | 0·12 (0·12-0·14) | 0·08 (0·08-0·11) | 0·09 (0·08-0·12) |
| Race classification (multitask) | 0·12 (0·11-0·14) | 0·10 (0·09-0·13) | 0·09 (0·08-0·10) |
|  | Youden’s J statistic (95% CI) | | |
| Random weights | 0·16 (0·15-0·17) | 0·22 (0·21-0·24) | 0·12 (0·11-0·14) |
| ImageNet | 0·34 (0·33-0·36) | 0·41 (0·40-0·43) | 0·35 (0·33-0·37) |
| Disease detection (all patients) | 0·36 (0·35-0·37) | 0·38 (0·37-0·40) | 0·38 (0·37-0·40) |
| Disease detection (White patients) | 0·33 (0·32-0·35) | 0·38 (0·37-0·40) | 0·35 (0·33-0·37) |
| Race classification (single task) | 0·77 (0·76-0·78) | 0·78 (0·77-0·79) | 0·80 (0·79-0·81) |
| Race classification (multitask) | 0·75 (0·75-0·76) | 0·77 (0·76-0·78) | 0·79 (0·78-0·80) |

Performance is determined in a one-vs-rest approach for each racial group. TPR and FPR in subgroups are determined using a fixed decision threshold optimized over the whole patient population for highest Youden’s J statistic. For comparison, we also report the performance for single task and multitask neural networks trained specifically for classifying race, confirming the high accuracy reported in the literature.[^8^](https://paperpile.com/c/d9G4RW/ypQ7s)

Table S7. SPLIT for sex classification with ResNet-34 on CheXpert

|  | White | Asian | Black |
| --- | --- | --- | --- |
| Neural network backbone | AUC (95% CI) | | |
| Random weights | 0·68 (0·67-0·69) | 0·65 (0·63-0·66) | 0·70 (0·68-0·72) |
| ImageNet | 0·91 (0·90-0·91) | 0·89 (0·88-0·90) | 0·91 (0·90-0·92) |
| Disease detection (all patients) | 0·91 (0·90-0·91) | 0·85 (0·84-0·86) | 0·91 (0·90-0·92) |
| Disease detection (male patients) | 0·91 (0·90-0·91) | 0·87 (0·86-0·88) | 0·90 (0·89-0·91) |
| Sex classification (single task) | 1·00 (1·00-1·00) | 1·00 (1·00-1·00) | 1·00 (1·00-1·00) |
| Sex classification (multitask) | 1·00 (1·00-1·00) | 1·00 (1·00-1·00) | 1·00 (1·00-1·00) |
|  | TPR (95% CI) | | |
| Random weights | 0·64 (0·59-0·68) | 0·55 (0·48-0·76) | 0·53 (0·49-0·76) |
| ImageNet | 0·83 (0·81-0·87) | 0·81 (0·78-0·86) | 0·88 (0·81-0·90) |
| Disease detection (all patients) | 0·83 (0·81-0·84) | 0·74 (0·72-0·81) | 0·80 (0·77-0·88) |
| Disease detection (male patients) | 0·84 (0·82-0·85) | 0·82 (0·75-0·86) | 0·84 (0·78-0·86) |
| Sex classification (single task) | 0·99 (0·98-0·99) | 0·98 (0·97-0·99) | 0·98 (0·98-0·99) |
| Sex classification (multitask) | 0·98 (0·97-0·98) | 0·97 (0·96-0·98) | 0·98 (0·96-0·99) |
|  | FPR (95% CI) | | |
| Random weights | 0·37 (0·32-0·41) | 0·33 (0·26-0·54) | 0·25 (0·20-0·47) |
| ImageNet | 0·17 (0·16-0·22) | 0·18 (0·15-0·23) | 0·21 (0·15-0·23) |
| Disease detection (all patients) | 0·17 (0·15-0·19) | 0·19 (0·17-0·26) | 0·13 (0·10-0·21) |
| Disease detection (male patients) | 0·19 (0·16-0·19) | 0·24 (0·17-0·28) | 0·20 (0·14-0·23) |
| Sex classification (single task) | 0·02 (0·01-0·02) | 0·03 (0·01-0·03) | 0·01 (0·01-0·02) |
| Sex classification (multitask) | 0·02 (0·02-0·02) | 0·03 (0·02-0·04) | 0·02 (0·01-0·04) |
|  | Youden’s J statistic (95% CI) | | |
| Random weights | 0·27 (0·26-0·28) | 0·22 (0·20-0·25) | 0·29 (0·26-0·32) |
| ImageNet | 0·66 (0·65-0·66) | 0·63 (0·61-0·65) | 0·67 (0·64-0·70) |
| Disease detection (all patients) | 0·66 (0·65-0·67) | 0·55 (0·53-0·57) | 0·67 (0·65-0·70) |
| Disease detection (male patients) | 0·66 (0·65-0·67) | 0·58 (0·56-0·60) | 0·64 (0·61-0·67) |
| Sex classification (single task) | 0·97 (0·97-0·97) | 0·96 (0·95-0·97) | 0·97 (0·96-0·98) |
| Sex classification (multitask) | 0·96 (0·96-0·96) | 0·94 (0·93-0·95) | 0·95 (0·94-0·97) |

Performance for classifying sex reported separately for each racial group. TPR and FPR in subgroups are determined using a fixed decision threshold optimized over the whole patient population for highest Youden’s J statistic. For comparison, we also report the performance for single task and multitask neural networks trained specifically for sex classification, confirming high accuracy reported in the literature.[^7^](https://paperpile.com/c/d9G4RW/SIlro)
