# Supplementary material for: Algorithmic encoding of protected characteristics in chest X-ray disease detection models
Source: eBioMedicine. 2023 Feb 13;89:104467. doi: 10.1016/j.ebiom.2023.104467 (PMC10025760; doi:10.1016/j.ebiom.2023.104467)
Supplement: Supplementary Figs. S1–S3 [file mmc3.docx]

# Supplementary material C: Additional figures


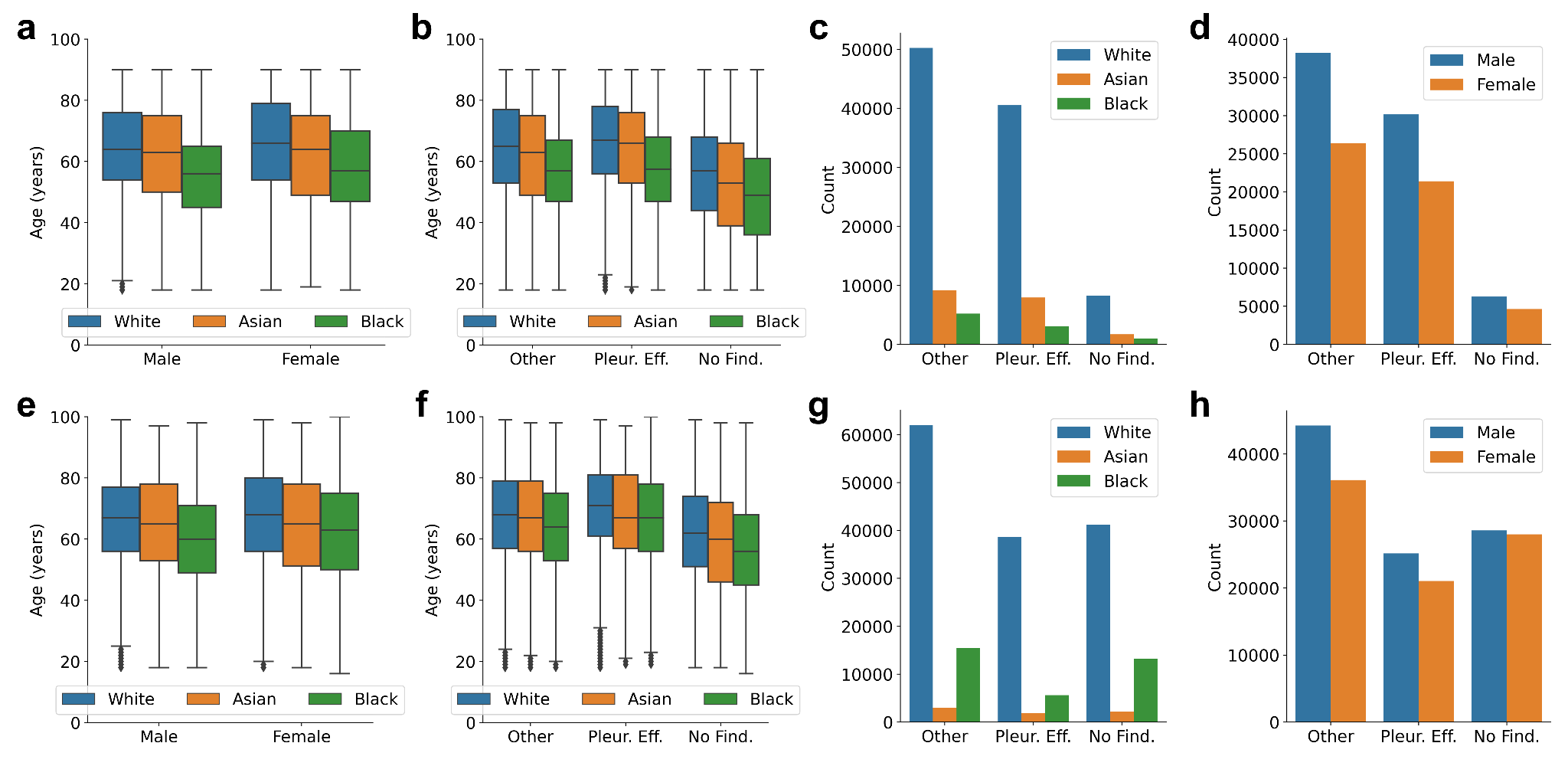


Figure S1. **Population characteristics of the original datasets.
a-d** CheXpert data shown at the top. **e-h** MIMIC-CXR shown at the bottom. **a,e** Age distribution over racial identity grouped by biological sex. **b,f** Age distribution over racial identity grouped by presence of disease. **c,g** Number of scans for each race grouped by presence of disease. **d,h** Number of scans for biological sex grouped by presence of disease. Whiskers in the box plot correspond to the largest and smallest samples inside the 1·5 interquartile range. Samples outside the boundary of the whiskers are plotted as outliers.


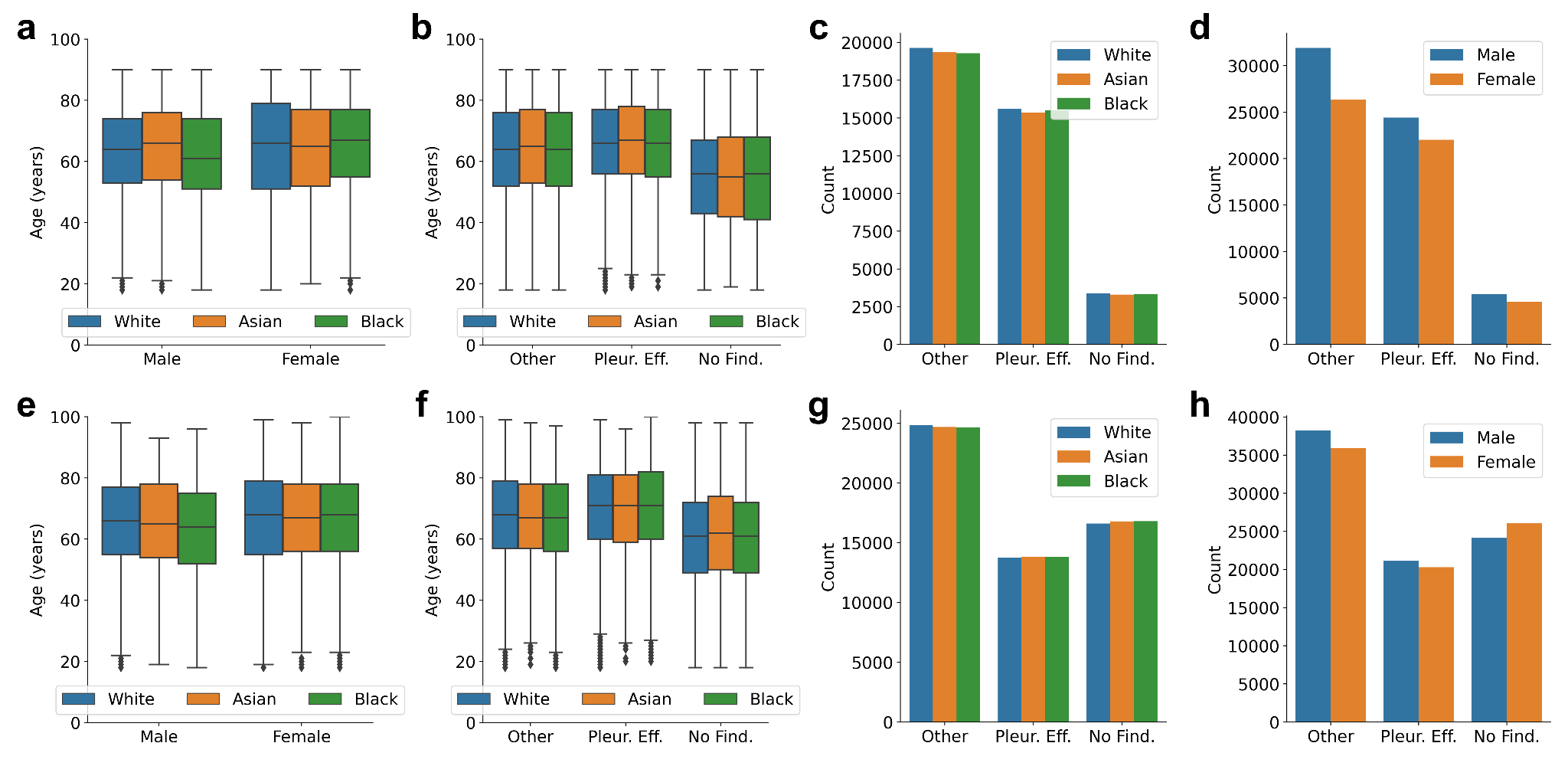


Figure S2. **Population characteristics of the resampled test-sets.
a-d** CheXpert data shown at the top. **e-h** MIMIC-CXR shown at the bottom. **a,e** Age distribution over racial identity grouped by biological sex. **b,f** Age distribution over racial identity grouped by presence of disease. **c,g** Number of scans for each race grouped by presence of disease. **d,h** Number of scans for biological sex grouped by presence of disease. Whiskers in the box plot correspond to the largest and smallest samples inside the 1·5 interquartile range. Samples outside the boundary of the whiskers are plotted as outliers.


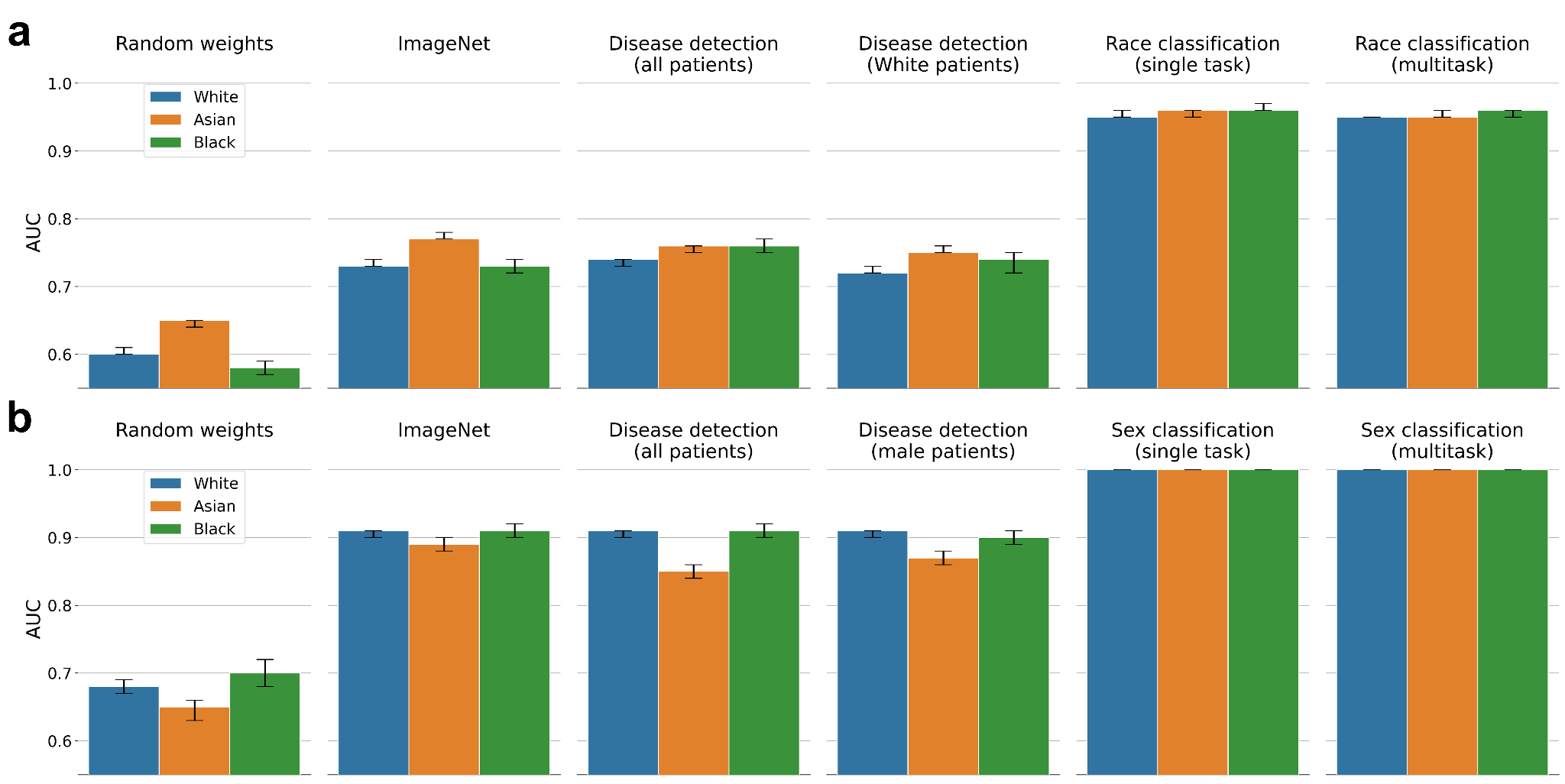


Figure S3. **Race (a) and sex (b) classification with ResNet-34 on CheXpert.**Race classification performance is determined in a one-vs-rest approach for each racial group. The first four columns are the race/sex classification results for SPLIT using different neural network backbones. Column five and six correspond to results from a single task race/sex classification model and the multitask model trained jointly for disease, sex, and race classification. SPLIT performance on the ImageNet backbone and disease detection backbones is very similar.
